# Bifunctional Temperature and Oxygen Dual Probe Based on Anthracene and Europium Complex Luminescence

**DOI:** 10.3390/ijms232314526

**Published:** 2022-11-22

**Authors:** Diogo Alves Gálico, Italo Odone Mazali, Fernando Aparecido Sigoli

**Affiliations:** 1 Institute of Chemistry, University of Campinas, UNICAMP, P.O. Box 6154, Campinas 13083-970, Sao Paulo, Brazil; 2 Department of Chemistry and Biomolecular Sciences, University of Ottawa, Ottawa, ON K1N 6N5, Canada

**Keywords:** lanthanides, anthracene, luminescent probes, thermometry, oxygen, polymer

## Abstract

In this work, we synthesized a polydimethylsiloxane membrane containing two emitter groups chemically attached to the membrane structure. For this, we attached the anthracene group and the [Eu(bzac)_3_] complex as blue and red emitters, respectively, in the matrix via hydrosilylation reactions. The synthesized membrane can be used as a bifunctional temperature and oxygen ratiometric optical probe by analyzing the effects that temperature changes and oxygen levels produce on the ratio of anthracene and europium(III) emission components. As a temperature probe, the system is operational in the 203–323 K range, with an observed maximum relative sensitivity of 2.06% K^−1^ at 290 K and temperature uncertainties below 0.1 K over all the operational range. As an oxygen probe, we evaluated the ratiometric response at 25, 30, 35, and 40 °C. These results show an interesting approach to obtaining bifunctional ratiometric optical probes and also suggest the presence of an anthracene → europium(III) energy transfer, even though there is no chemical bonding between species.

## 1. Introduction

The determination of temperature and oxygen levels is extremely important for numerous fields, such as biology, food engineering, environmental analysis, and aerospace fields [1,2,3,4,5,6,7,8,9,10,11,12]. Due to their importance in several processes, the development of fast, reliable, versatile, and remote probes is highly desirable. As stated by Wang and Wolfbeis [13], a sensor is a miniaturized probing device able to deliver real-time information on the concentration level of a specific physical or chemical parameter. A sensor device is composed of four main parts: the sensitive probe (for example, an emitter species whose optical properties are affected by the presence of an analyte or with the temperature change), a host or matrix (frequently a polymeric film/membrane) for hosting the probing species, an electronic read-out system, and a device for data processing [13]. Among several possibilities, using an optical probe has the advantage of enabling non-invasive probing, an easy miniaturization, and the possibility of being used for imaging ranging from micro to large scales [14,15,16,17].

Lanthanide(III) ions are among the most promising systems for optical probes due to the intrinsic characteristics of the *4f-4f* transitions, such as sharp emission lines and long emission lifetimes [18,19,20]. There are a plethora of examples demonstrating the use of trivalent lanthanide ions as sensitive probes, as, for example, for the probing of pH [21,22,23,24,25,26], cations [27,28,29,30], anions [31,32,33,34], explosives [35,36,37,38], reactive oxygen species (ROS) [39,40], molecular oxygen [41,42,43,44,45,46,47], temperature [48,49,50,51,52,53,54,55,56,57,58,59,60], among other analytes. 

When dealing with optical probes, it is possible to implement different probing strategies. For example, there are several examples using single band intensities [61,62,63], band-shift [64,65,66], emission lifetime [67,68,69], excited-state absorption [70,71], and single/dual band ratiometric [72,73,74,75,76,77] probing approaches. The latter consists of monitoring the analyte effect on the ratio of two distinct emission bands. With this probing strategy, it is possible to overcome drawbacks arising from the optoelectronic oscillations and the material inhomogeneities. Moreover, the detection scheme is easier than the one needed for measurements on the time-domains (lifetime, for example).

Over the last few years, our group has been developing a strategy that allows us to manipulate and modulate the two most important parts of an optical sensor, the sensitive probe, and the hosting matrix [78,79,80,81]. We have been focusing our attention on polydimethylsiloxane (pdms) a polymeric matrix chemically modified via hydrosilylation reaction, which allows us to chemically bond any organic or inorganic group containing vinyl and allyl groups [82]. The use of pdms as the polymeric matrix relies on the high oxygen permeability, biocompatibility, hydrophobic character, and excellent adhesion properties [83,84].

For the sensitive probe, several compounds can be chemically attached to the matrix, but here we are aiming for the attachment of a compound of which the optical properties are highly sensitive to the oxygen level and a second compound that shows a strong emission dependence with the temperature. To achieve our intended goal, we put our efforts into attaching the anthracene group as an oxygen-sensitive blue emitter moiety [85] and the red emitter [Eu(bzac)_3_] complex due to the well-known temperature-dependent emissive properties of this complex [86].

**Scheme 1:**
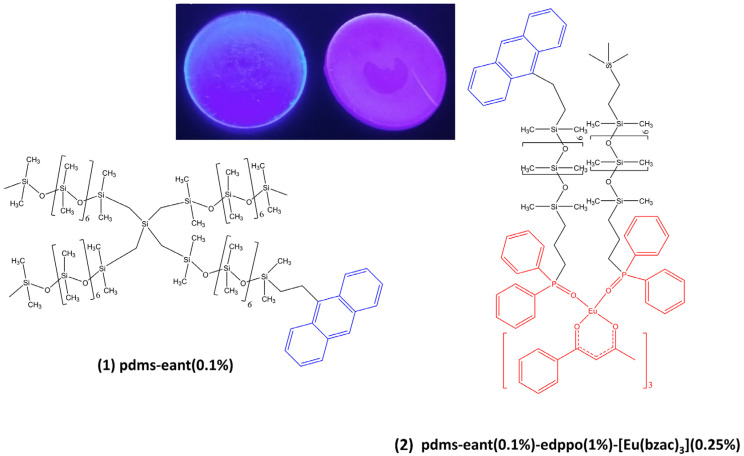
Schematic depiction of the structures for membrane **(1)** and **(2)**. Inset: Membranes under a 365 nm excitation.

With the target goal of developing a ratiometric temperature and oxygen dual optical probe, two different polymeric membranes were synthesized. The first one, only containing the blue emitting anthracene group chemically bonded in the matrix, was used for control purposes, **(1)** pdms-eant(0.1%), and the second, containing the anthracene group and the red emitting europium(III) complex, **(2)** pdms-eant(0.1%)-edppo(1%)-[Eu(bzac)_3_](0.25%) (Scheme 1).

## 2. Results and Discussion

**Figure 1:**
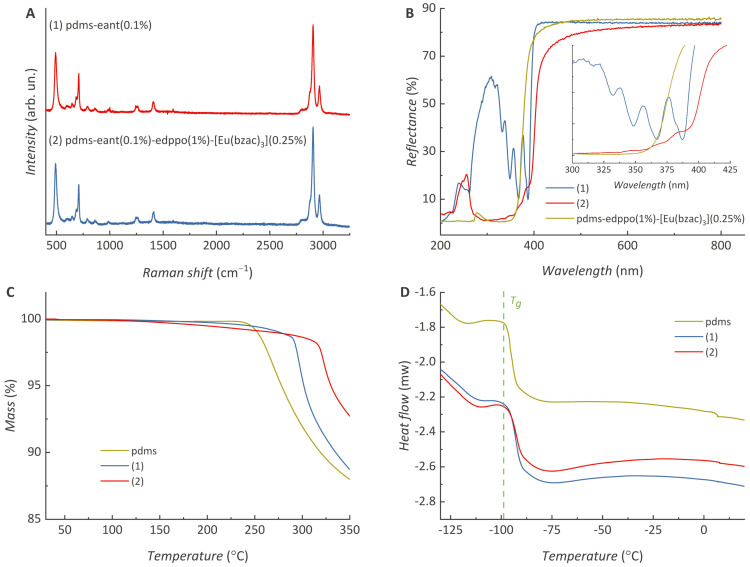
(**A**) Raman spectra for membranes **(1)** and **(2)**. (**B**) DRS spectra for membranes **(1)**, **(2)**, and pdms-edppo(1%)-[Eu(bzac)_3_](0.25%) (from Ref. [78]). (**C**) TGA and (**D**) DSC curves for a pdms membrane and membranes **(1)** and **(2)**.

In recent works, our group demonstrated a methodology to chemically bond allyl and vinyl groups in a pdms matrix via hydrosilylation reactions [78,79,80,81]. Details related to the attachment mechanism can be found in these previously published works. Herein, we are attaching a vinylanthracene group as a blue emitter and an europium(III) complex as a red emitter. The Raman spectra for membranes **(1)** and **(2)** are shown in Figure 1A. The most important observed feature is the absence of the νSi-H band at approximately 2130 cm^−1^. This absence clearly indicates that the functionalization of the membrane with the anthracene group was fully achieved [78,79,80,81]. The two synthesized membranes are transparent in the visible region of the electromagnetic spectrum (Figure 1B), and membrane **(1)** emits the characteristic blue emission from the anthracene group under 365 nm excitation, while membrane **(2)** emission entails a mixture of anthracene and europium(III) components (Scheme 1, discussion below). The diffuse reflectance spectrum (DRS) for membrane **(1)** reveals the characteristic anthracene absorptions (Figure 1B), with the vibrational components [87]. DRS spectrum for membrane **(2)** shows the characteristic absorptions from bzac and edppo ligands [78,86], but also present the vibrational structure from anthracene, which overlaps the [Eu(bzac)_3_] complex absorption (Figure 1B, inset).

The TGA curves for membranes **(1)** and **(2)** are shown in Figure 1C. These data indicate that the membranes are thermically stable until up to 155 and 192 °C, respectively. The DSC curves for membranes **(1)** and **(2)** are shown in Figure 1D. It is evident from this data that the membrane functionalization with the anthracene group does not change the glass transition of the polymeric matrix (~−98 °C for pdms). The previously shown data strongly suggests that the attachment of the anthracene moiety was effectively attained for membrane **(1)** and both groups for membrane **(2)**. 

**Figure 2:**
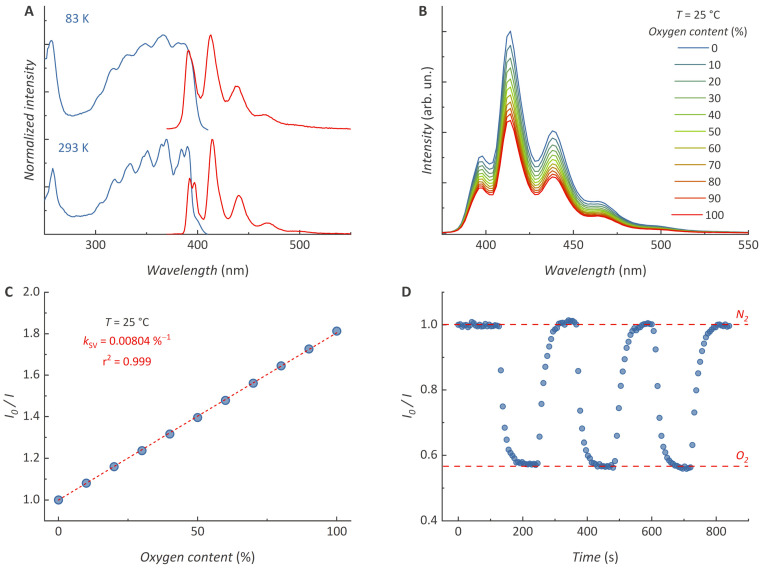
(**A**) Excitation (blue lines; λ_em_ = 414 nm) and emission (red lines; λ_ex_ = 350 nm) spectra for membrane **(1)** at 83 and 293 K. (**B**) Oxygen dependence of the emission for the membrane **(1)** at 25 °C (λ_ex_ = 350 nm). (**C**) Stern–Volmer plot obtained via the integration of the whole anthracene emission band (380–550 nm range). (**D**) Atmosphere cycling (λ_ex_ = 350 nm; λ_em_ = 414 nm) for membrane **(1)** at 25 °C.

With the attachment of the emissive groups, we started evaluating the membranes as optical probes. We first started analyzing membrane **(1)** as a control membrane with the goal of understanding the spectroscopic properties of the anthracene group attached to the pdms membrane. Excitation and emission spectra at 293 ad 83 K for membrane **(1)** are shown in Figure 2A and reveal the characteristic and expected vibrational structure of the anthracene group [87]. The emission spectra are centered in the blue range, as expected for anthracene. Oxygen dependence of the membrane **(1)** was studied at 25 °C as a control (Figure 2B), and it is possible to note that anthracene emission is quenched with an increasingly large amount of oxygen. For these measurements, the membrane was cut into 10 × 10 mm squares and placed inside a TS1500 stage (Linkam) coupled to a mass flow controller (MFC) device (see Materials and Methods section). The N_2_ and O_2_ gases were mixed within the MFC device (with a constant volume of 1 mL/min), varying from 0% to 100% of O_2_. The Stern–Volmer plot at Figure 2C shows an *I*_0_/*I* value of 1.81 at 25 °C and a Stern–Volmer constant (*K_SV_*) calculated by Equation (1) (see Materials and Methods section) of 0.00804%^−1^. The O_2_↔N_2_ cycle data for membrane **(1)** is shown in Figure 2D and confirms the stability and reversibility for membrane **(1)**. A response time (N_2_→O_2_) of approximately 50 s and a recover time (O_2_→N_2_) of approximately 70 s were obtained. With these data, we have been able to evaluate how the O_2_ levels impact the optical properties of the control membrane **(1)**. 

As our target goal is the development of ratiometric bifunctional temperature and oxygen probes, we turned our attention to membrane **(2)** containing the blue emitter anthracene and the red emitter europium(III) groups.

**Figure 3:**
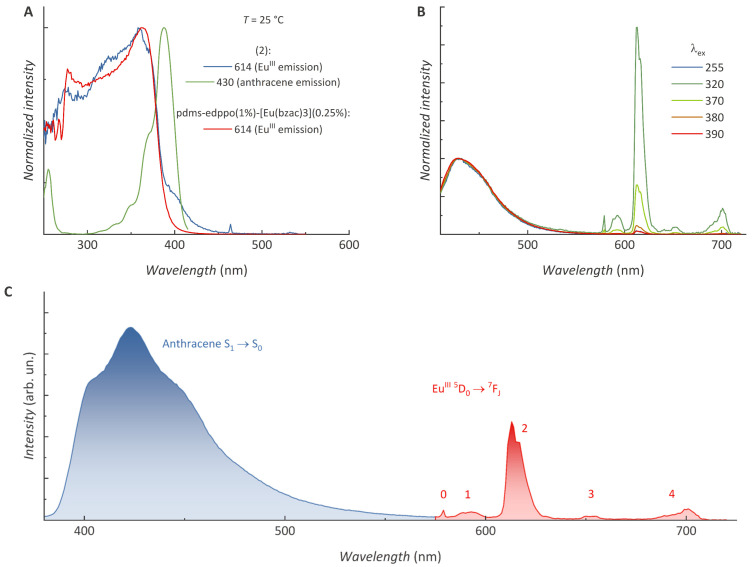
(**A**) Excitation spectra of the membrane **(2)** and pdms−edppo(1%)− [Eu(bzac)_3_](0.25%) membrane (from Ref. [78]). (**B**) Emission spectra for membrane **(2)** with different excitation wavelengths. These spectra were collected starting at 410 nm due to the proximity with some of the excitation wavelengths and were normalized by the maximum of the anthracene component. (**C**) Emission spectrum for membrane **(2)** (λ_ex_ = 370 nm). Red numbers refer to the J sublevel from europium(III).

Excitation spectra for membrane **(2)**, monitoring both the anthracene (blue component) and the europium(III) (red component) emissions and the spectra for the pdms−edppo(1%)− [Eu(bzac)_3_](0.25%) membrane (from Ref. [78]) obtained at 25 °C are shown in Figure 3A. Although it is not the goal of this work, the different excitation profile allows us to modulate the emission spectrum accordingly to the excitation energy (Figure 3B), suggesting the potential of this system for anti-counterfeiting applications [88,89,90]. It is also possible to note that even though not chemically bonded to the europium(III) ion, the anthracene moiety is able to contribute to the europium(III) sensitization as we can observe the anthracene component on the excitation spectrum of **(2)** when monitoring the europium(III) emission (blue line; Figure 3A). This is the first indication for a possible anthracene → europium(III) ET in this system (discussed below).

The emission spectrum for membrane **(2)** with excitation at 370 nm is shown in Figure 3C. It is possible to observe the presence of the anthracene emission in the 380–565 nm range and the characteristics of the europium(III) components arising from the ^5^D_0_ emitter state.

**Figure 4:**
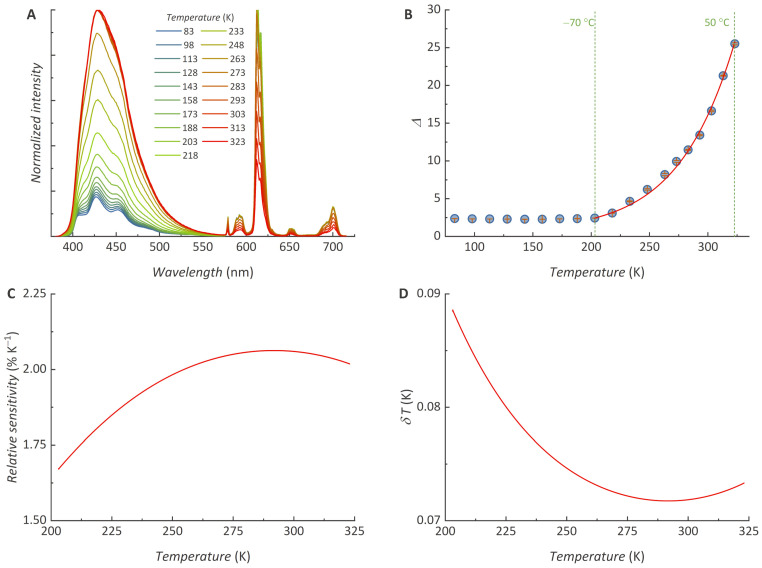
(**A**) Temperature dependence of the emission spectra membrane **(2)** (λ_ex_ = 370 nm). (**B**) Temperature dependence of the thermometric parameter. (**C**) Calculated relative sensitivity, and (**D**) calculated temperature uncertainty.

In order to prove the bifunctionality of **(2)**, we first studied the temperature dependence of the emission spectra in the 83–323 K range. As can be observed in Figure 4A, at low temperatures, europium(III) components dominate the spectra, while at higher temperatures, anthracene emission shows higher intensities. Due to this change, the present membrane can be easily applied as a ratiometric temperature probe. For the probing strategy, we selected the ratio between the integrated area of the anthracene emission band (380–560 nm range) and the ^5^D_0_ → ^7^F_2_ transition band (605–640 nm range) of the europium(III) complex to be used as the thermometric parameter (*Δ*). The temperature evolution of *Δ* (Figure 4B) reveals that from 83 to approximately 203 K, no significant changes occur on *Δ* values, so we started the analysis above this temperature. We have been able to apply a Boltzmann-like function (Equation (2), see Materials and Methods section) as implemented in OriginPro 2021 to fit the experimental data. Applying Equation (3), we were able to obtain the relative thermal sensitivity (*S_R_*) for **(2)** with a maximum value of 2.06% K^−1^ at 290 K and an operational range between the 203–323 K range (Figure 4C). The system shows temperature uncertainty (*δT*) below 0.1 K over all the operational range (Figure 4D), thus highlighting the potential of **(2)** as a luminescence thermometer.

With the success in demonstrating the potential of **(2)** as a temperature probe, we turned our attention to the effect of the oxygen level on the emissive properties. However, it should be noted that the previously discussed temperature dependence of the emission may be detrimental for its use as an oxygen probe since temperature is known as the biggest source of error in optical oxygen sensors [91]. Thus, it is of huge importance to understand how the temperature affects the oxygen probing in order to develop the required correction strategies. Although these corrections can also be valuable when aiming the development of an optical probe for the simultaneous probing of O_2_ and temperature, here we are only interested in showing the oxygen dependence at different temperatures and proving the potential of the system as bifunctional optical probe.

**Figure 5:**
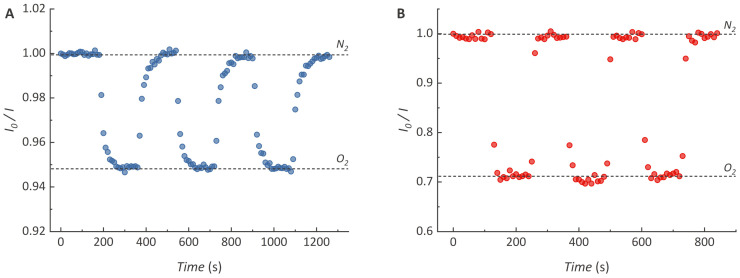
Oxygen and nitrogen cycles for (**A**) anthracene (λ_ex_ = 360 nm; λ_em_ = 414 nm) and (**B**) europium(III) emissions (λ_ex_ = 360 nm; λ_em_ = 614 nm) of the membrane **(2)** at 25 °C.

For the oxygen probing experiments, we excited membrane **(2)** at 360 nm, and the same integrated areas used for the temperature probing measurements were selected. At 25 °C, it is evident that both emissions are affected and diminished as a function of an increasing oxygen concentration (Figure 5A,B). The anthracene emission shows a decrease of approximately 5%, while the europium(III) emission is reduced by approximately 30%. This is an interesting result, since for membrane **(1)**, which contains the same amount of anthracene, the emission quenching is much more pronounced (Figure 2B,D). The system is stable and reversible, and the response time (N_2_→O_2_) is approximately 50 and 20 s for the anthracene and europium(III) emissions, respectively, while the recovery time (O_2_→N_2_) is approximately 100 and 30 s.

**Figure 6:**
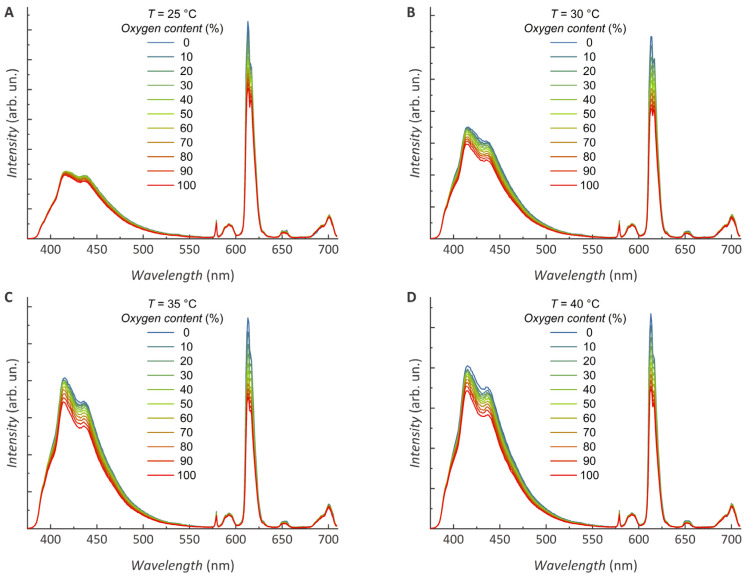
Oxygen dependence of the emission for the membrane **(2)** at (**A**) 25, (**B**) 30, (**C**) 35, and (**D**) 40 °C (λ_ex_ = 360 nm).

**Figure 7:**
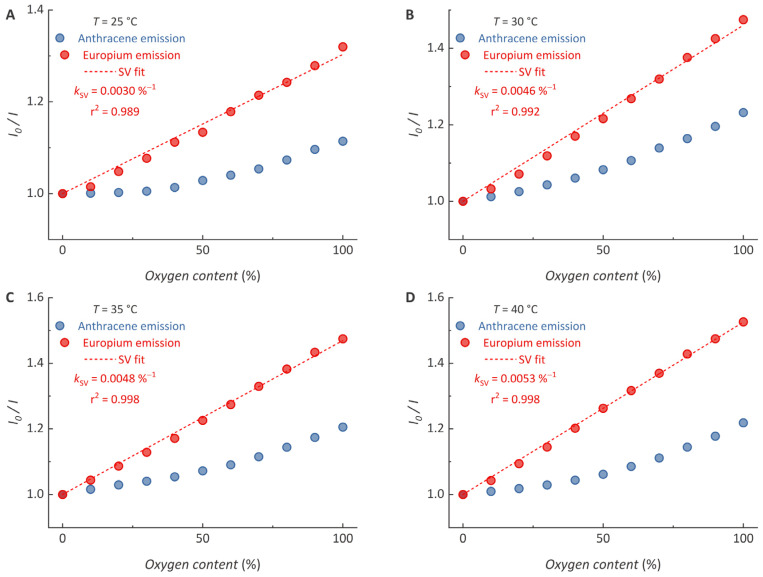
Stern–Volmer plot for the membrane **(2)** at (**A**) 25, (**B**) 30, (**C**) 35, and (**D**) 40 °C.

It is possible to notice that at higher temperatures the same behavior is observed, with both emissions being quenched by an increasing oxygen concentration (Figure 6). The Stern-Volmer plots for membrane **(2)** are shown in Figure 7, revealing that the europium(III) emission follows a linear Stern–Volmer behavior. Thus, a homogeneous quenching of the europium(III) emission occurs in membrane **(2)**. The Stern–Volmer constant was calculated for the europium(III) emission at different temperatures by using Equation (1) (see Materials and Methods section), revealing an increase from 0.00303%^−1^ at 25 °C to 0.00536%^−1^ at 40 °C. This increase of the constant at higher temperatures confirms the presence of a dynamical (collisional) quenching [92]. An intriguing aspect from Figure 7 is that the oxygen dependence for the anthracene emission cannot be modeled by a Stern–Volmer relation in membrane **(2)**. This different behavior suggests the presence of a different operative mechanism on the anthracene emission for membrane **(2)** as for membrane **(1)** the anthracene emission quenching follows the Stern–Volmer relation (Figure 2C), being a second indication of the presence of the anthracene→europium(III) ET. Herein, we can hypothesize that since the energy transfer from an organic moiety to a lanthanide(III) ion occurs much faster [93,94] than the observed oxygen quenching, the most probable explanation for the decrease of the anthracene oxygen quenching is that a significant part of the energy was transferred to europium(III) before being quenched by oxygen.

In order to implement a ratiometric strategy for the oxygen probing, we selected the ratio (*R*) between *I*_1_ (anthracene emission, 380–560 nm range) and *I*_2_ (europium emission, 605–640 nm range) for our analysis.

**Figure 8:**
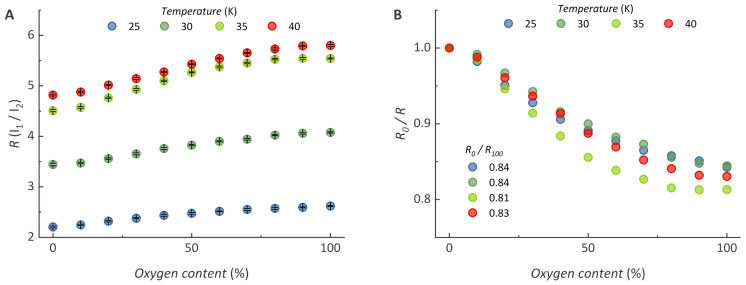
Variation of the (**A**) *R* (*I*_1_/*I*_2_) and (**B**) *R_0_/R* with the oxygen content at different temperatures.

The oxygen dependence for *R* at different temperatures is shown in Figure 8A as well the *R*_0_/*R* plot (Figure 8B). The present data clearly suggest that the strategy herein adopted for membrane (2) is appropriate and very promising when aiming a ratiometric optical probing for oxygen. 

Even though the observed variation for *R*, with the oxygen concentration being small, the probing strategy here demonstrated is extremely promising since the use of lifetime and/or single band strategies is dominating for oxygen optical probes, and the possibility of binding other compounds with different sensitivities in the pdms matrix opens new perspectives on the development of new materials for multifunctional sensing. This approach is easily adaptable, with the possibility of attaching different complexes and/or organic groups, it is easy to synthesize, shows high stability and reversibility, and the chemical attachment of the emitting species into the polymeric matrix reduces the possibility of the probe lixiviation during real-life application in different environments.

In summary, we demonstrate that the functionalization via chemical bonding of anthracene (blue emitter) and the [Eu(bzac)_3_] complex (red emitter) in a pdms matrix allows us to develop a bifunctional ratiometric probe for temperature and oxygen. By using the ratio between the anthracene emission and europium(III) ^5^D_0_ → ^7^F_2_ transition band integrated areas, we accessed a bifunctional ratiometric probe. For the temperature probing, an operational range of 203 to 323 K is observed, with a maximum *S_R_* of 2.06% K^−1^ at 290 K. Temperature uncertainties below 0.1 K were observed over the entire operational range. For the oxygen probe, we analyzed the oxygen dependence of the ratio between anthracene and europium(III) emission components at 25, 30, 35, and 40 °C. The system shows different *R*_0_/*R* responses at different temperatures, indicating the necessity of temperature corrections when aiming for simultaneous temperature and oxygen optical probes.

Although not chemically bonded, two pieces of evidence strongly support the presence of an anthracene → europium(III) energy transfer operating on membrane **(2)**. We foresee that a deep study modulating the concentration of anthracene and europium(III) complex in a similar fashion can result in highly modulable materials with different temperature and oxygen sensitivities due to different energy-transfer efficiencies. 

The approach herein demonstrated is very promising and adaptable since the attachment of different organic and inorganic emitters to the matrix is possible, and consequently, this opens new avenues for obtaining multifunctional optical probes.

## 3. Materials and Methods

### 3.1. Membrane Synthesis

The synthesis of [Eu(bzac)_3_(H_2_O)] was carried out as previously reported [86]. In summary, the complex was prepared by a stoichiometric reaction between an ethanolic solution of sodium benzoylacetonate and EuCl_3_·6H_2_O. A 0.1 mol L^−1^ NaOH aqueous solution was used to adjust the pH to 6.5. The solution was refluxed for 4 h, the pale-yellow precipitate filtered, washed with deionized water, and dried at room temperature under reduced pressure.

The synthesis of the membranes follows similar procedures to those previously reported [78] and is described in detail below:

For the synthesis of membrane **(1)**, 1.0 g of pdms-H (hydride-terminated polydimethylsiloxane, MW = 580 g mol^−1^) and 0.1% of 9-vinylanthracene (related to the % of pdms-H Si-H bonds) were solubilized in 2.0 mL of anhydrous toluene in a silanized three-neck round-bottom flask. Then, three drops of Karsted catalyst were added, and the solution was stirred for 2 h at room temperature under an N_2_ atmosphere. After this time, 99.9% (in relation to the % of Si-H bonds) of the tetravinylsilane (TVS) cross-linker and three drops of Karsted catalyst were added. After 2 min of stirring, we quickly transferred the solution to a 1.0 mm depth, 50 mm diameter circular PTFE template to yield a self-supported membrane.

For the synthesis of membrane **(2)**, 1.0 g of pdms-H, 1.0% of allyldiphenylphosphine oxide (adppo), and 0.1% of 9-vinylanthracene (amount related to the % of Si-H bonds) were solubilized in 2.0 mL of anhydrous toluene in a silanized three-neck round-bottom flask. Then, three drops of Karsted catalyst were added, and the solution was stirred for 2 h at room temperature under N_2_ atmosphere. Then, 0.25% of the europium(III) complex (related to the % of Si-H bonds) was added and the solution was stirred for 12 h at room temperature under an N_2_ atmosphere. After this time, 98.9% of the TVS cross-linker (related to the % of Si-H bonds) and three drops of Karsted catalyst were added. After 2 min of stirring, we quickly transferred the solution to a 1.0 mm depth, 50 mm diameter circular PTFE template to yield a self-supported membrane. 

The membranes were dried under vacuum for 2 days before the measurements. For all the optical measurements the membranes were cut into 10 × 10 mm squares in order to fit into the cryostat and MFC sample holders.

### 3.2. Characterizations

Thermogravimetric (TGA) curves were obtained with a SDT 2960 system (TA Instruments) using an air flow of 100 mL/min and a heating rate of 10 °C/min. Differential scanning calorimetry (DSC) analysis was performed with a DSC 1 system (Mettler Toledo) using a heating rate of 10 °C/min and a nitrogen flow of 100 mL/min. 

Raman spectroscopy was performed on a T64000 (Horiba Jobin-Yvon) using a 632.8 nm He/Ne laser and a spectral resolution of 2 cm^−1^. 

Diffuse reflectance spectra (DRS) were obtained with a Varian Cary 5000 spectrophotometer in the 200–800 nm range.

Photoluminescence studies were performed with a Fluorolog-3 spectrofluorometer (Horiba FL3-22-iHR320). An ozone-free Xe lamp (Ushio, 450 W) was employed as the excitation source. All the spectra were corrected according to the instrument response. All the luminescence measurements were obtained in triplicate.

For the temperature-dependent luminescence spectra, samples were placed in a cryostat (Janis Research Company VNF-100).

For the atmosphere-dependent studies, we used a digital mass flow controller (Horiba STEC SEC-N100 Series) controlled by an xPH-100 Power Hub system. The samples were placed in a TS1500 stage (Linkam) coupled with a T95-HT temperature controller (Linkam).

### 3.3. Data Analysis

For the ratiometric analysis employed in this work, we used the ratio between *I*_1_ (anthracene emission, 380–560 nm range) and *I*_2_ (europium emission, 605–640 nm range).

Stern–Volmer equation (Equation (1)) was used to quantify the oxygen dependence of the luminescence [92]:

(1) $$\frac{I_{0}}{I}=1+K_{SV}\left[O_{2}\right]$$

where, *I*_0_ and *I* correspond to the emission intensities of the probe in the absence and in the presence of oxygen, respectively; *K_SV_* is the Stern–Volmer constant; and [*O*_2_] is the oxygen concentration, which can be expressed in oxygen molar concentration, percentage, or partial pressure.

For the optical thermometry analysis, the variation of the thermometric parameter, *Δ*, at the 203–323 K range can be modelled with a Boltzmann-like equation, as implemented in OriginLab 2021 (Equation (2)). It is important to note that here the equation is used only as a calibration function with no physical meaning.

(2) $$y=\frac{A_{1}-A_{2}}{1+e^{\left(x-x_{0}\right)/dx}}+A_{2}$$

The figure of merit of optical thermometers is the relative sensitivity (*S_R_*) expressed by Equation (3) [48]:

(3) $$S_{R}\left(\%\right)=\left[\left(\partial ∆/\partial T\right)/∆\right]\cdot 100$$

## Data Availability

All datasets generated for this study are included in the article and will be shared upon reasonable request to the corresponding author.
